# Continuation versus discontinuation of treatment for severe dementia: randomized, pragmatic, open-label, clinical trial to evaluate the efficacy of continuing drug treatment in patients with severe dementia (STOP-DEM)

**DOI:** 10.1186/s12877-019-1122-2

**Published:** 2019-04-11

**Authors:** Aina Soler, Guillem Amer, Alicia Leiva, Joana Ripoll, María Angeles Llorente, Alfonso Leiva, Joana Maria Taltavull, Rosa Molina, Joan Llobera

**Affiliations:** 1 Primary Care Research Unit of Mallorca, Baleares Health Services-IbSalut, Palma, Spain; 2 Instituto de Investigación Sanitaria de Palma, Palma, Spain; 3 Son Espases Hospital, Baleares Health Services-IbSalut, Palma, Spain; 4 Manacor Hospital, Baleares Health Services-IbSalut, Mallorca, Spain; 5 Son Pisa Health Care Centre, Baleares Health Services-IbSalut, Palma, Spain

**Keywords:** Dementia, Cholinesterase inhibitors, Deprescription, Randomized controlled trial

## Abstract

**Background:**

Previous observational studies and clinical trials have shown that cholinesterase inhibitors (with or without memantine) provide benefit for patients with mild-to-moderate Alzheimer’s disease. However, the impact of treatment continuation after progression to severe disease is unknown. The main aim of this study is to evaluate the effect and safety of continuing treatment with ChEIs (with or without memantine) for patients with severe dementia.

**Methods:**

This randomized, pragmatic, open-label clinical trial with blinded evaluators will evaluate the efficacy of continuing drug treatment in patients with advanced dementia. A total of 302 community-dwelling patients with severe dementia, Alzheimer’s disease, with or without a coexisting diagnosis of vascular dementia, and a score of 10 or less on the Mini-Mental State Examination who received previous treatment with a cholinesterase inhibitor (with or without memantine) for at least 3 months, will be randomized to continue or discontinue drug treatment. Follow-up will be 12 months or until the primary endpoint is achieved. The primary endpoint is entry into institutional care and progression of disability, defined as a loss of 2 of 4 basic functions, or 6 of 11 instrumental functions, according to the Bristol Activities of Daily Living Scale at 12 months. The secondary outcomes are patient changes in functional and cognitive state, quality of life, and caregiver burden.

**Discussion:**

We expect that the results of our study will allow to identify if there is clinical relevant impact for patients and caregivers between maintaining or halting pharmacological treatment.

**Trial registration:**

The study was prospectively registered in the REec (2017–000042-22) on May 11 2017 and ID ISRCTN12134230 on February 25 2019.

## Background

Dementia is a chronic progressive mental disorder that adversely affects higher cortical functions, including cognition and behavior leading up to disability and dependence in daily life activities. It has become a major public health concern because of its increasing prevalence, chronicity, burden for caregivers, and the high personal and financial costs needed for care. Age is the main risk factor for dementia, and its prevalence in Spain is 5 to 14.9% in people over 65 years-old, and 9 to 17.2% in people over 70 years-old [1]. Thus, Alzheimer’s disease (AD) and other dementias are currently considered a major public health concern. A recent forecast predicted 14 million cases in Europe by 2050 [2]. In Spain, up to 11% of deaths overall are related to dementias, and about one-third of people older than 85 years die from dementia [3].

There are no cures for AD, but most guidelines recommend pharmacological treatment to alleviate symptoms and might delay disease progression. Cholinesterase inhibitors (ChEI; donepezil, galantamine, rivastigmine, and tacrine) are approved for managing mild to moderate AD. Memantine [an N-methyl D-aspartate (NMDA) receptor antagonist] is approved for treatment of moderate to severe dementia of the Alzheimer’s type but these drugs are also given off-label for other types of dementia (vascular and mixed dementias), and severe dementia. The pharmacotherapeutic agents available to treat problems associated with dementias have different levels of evidence to support their efficacy. ChEIs produce small, short-term improvements in the cognitive function of patients with mild to moderate dementia, which can result in statistically significant changes, but only marginally significant clinical improvements in cognition and global assessment scores. Moreover, these drugs provide minimal benefits for those who have severe disease, are receiving long-term treatment, and who are elderly [4]. There is very limited evidence to guide the difficult decision about whether to continue or discontinue treatment as disease progresses.

ChEIs can also cause adverse effects, and these increase with ChEI dose. Adverse effects are also 2-fold greater in those over 85 years-old than in younger patients. In particular, cholinergic stimulation leads to a 2- to 5-fold increased risk for gastrointestinal, neurological, and cardiovascular adverse effects, the most serious being weight loss, debility, and syncope [5].

Some clinical trials have evaluated the efficacy and safety of different drug treatments for patients with dementia. The first of these was the AD2000 study [6]. These authors used a double-blind procedure to examine the effect of the dose and duration of donepezil treatment on disability, dependency, psychological factors and behavioral symptoms of dementia, welfare of the caregiver, and delay in institutionalization. They recruited 565 patients with mild or moderate AD (in the initial phase) and randomized them to receive donepezil (5 or 10 mg daily) or placebo for 12 weeks. The 486 patients who completed this phase of the study were then randomized again to receive donepezil or placebo. The main outcome measures were institutionalization and progression of disability, assessed by the Bristol activities of daily life scale (BADLS). After 3 years, the effects of donepezil and placebo were similar for institutionalization (42% vs. 44%) and progression of disability (58% vs. 59%). The treatment groups had a relative risk (RR) of institutionalization of 0.97 (95% confidence interval [CI]: 0.72–1.30, *p* = 0.8), an RR of progression of disability or institutionalization of 0.96 (95% CI, 0.74–1.24, *p* = 0.7). The two groups also had no differences in the symptoms of psychological and behavioral dementia (SPBD), costs of formal care, adverse events (AE), or mortality. It is important to highlight that these results differ from the clinical trials performed by the drug manufacturer, which examined patients in usual practice conditions, and included patients with vascular dementia (VD) and/or mixed dementia.

The DOMINO-AD clinical trial [7] evaluated the effectiveness of ChEIs (with or without memantine) in 295 patients with moderate-to-severe AD. All patients who received prior treatment with donepezil (minimum of 3 months) were randomly assigned to a group that continued or discontinued this drug (with or without memantine) for 1 year. Relative to those who stopped donepezil, patients who continued donepezil experienced less mental deterioration, based on the Mini–Mental State Examination (MMSE, *p* < 0.001) and the BADLS (*p* < 0.001) after 1 year. In addition, patients who took memantine scored better on both tests than those who did not (MMSE: *p* < 0.001; BADLS: *p* = 0.02). However, treatment with both drugs provided no greater benefit than donepezil alone. These results support the continued use of donepezil in patients with moderate-to-severe AD, based on evaluation of its functional benefits at 12 months [8].

A secondary outcome of this study was to evaluate whether continuation of pharmacological treatment would also delay time to institutionalization in this patients [7]. Although more than half of the patients entered a nursing home over the follow up period, a post-hoc analysis showed that continuation of a drug treatment delayed nursing home placement in patients with AD. Patients who continued on drug treatment had lower risk of entering a nursing home within the first year after intervention [hazard ratio (HR) 2.09 (95% confidence interval 1.29 to 3.39)], however there was no difference between continuation or discontinuation in the subsequent 3 years [HR 0.89 (0.58 to 1.35)].

A recently published meta-analysis of randomized controlled trials examined the effects of ChEI discontinuation in patients with AD. As previous studies, the results suggested that ChEI discontinuation may be associated with a statistically significant deterioration in cognition and neuropsychiatric symptoms but, again only one study included patients with advanced dementia (MMSE < 10) [9].

Importantly, these studies only examined patients with AD and very few studies evaluated the efficacy of these drugs in patients with other types of dementia or severe dementia. Thus, the specific aim of the present study of patients with advanced dementia is to compare the effect of maintaining treatment with a ChEI (with or without memantine) vs. discontinuing drug treatment on the time to institutionalization and/or progression of disability (defined as a loss of 2 of 4 basic functions, or 6 of 11 instrumental functions, according to the BADLS) at 12 months.

Even if there is considerable uncertainty on the benefits and harms of both prescribing and deprescribing ChEI, most guidelines recommend individualized treatment decisions depending on patient and caregiver or family experiences. The only relevant domains identified by most authors are a lack of response or a loss of effectiveness, which can be difficult to evaluate in patients with advanced dementia [10, 11]. Well-designed, long-term studies of discontinuation have not been conducted; such evidence is needed to provide a scientific basis for practice guidelines [12].

The main aim of this study is to evaluate the effect and safety of continuing treatment with ChEIs (with or without memantine) for patients with severe dementia.

## Methods

### Design

**Fig. 1:**
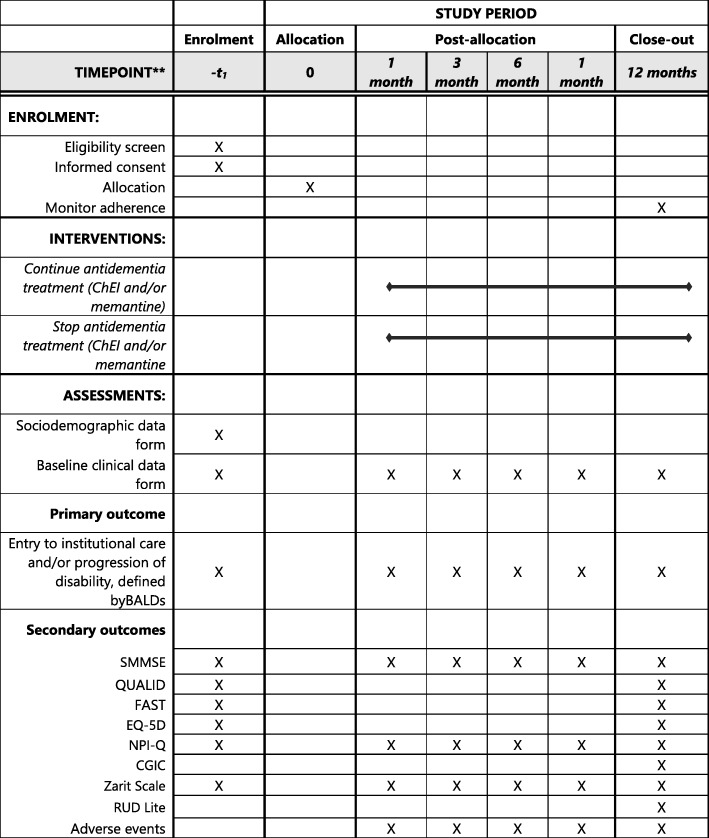
Schedule of enrolment, interventions, and assessments

This study of patients with advanced dementia is a clinical, pragmatic, multicenter, open trial with blinded assessors and a parallel randomized design that will evaluate the effect of maintaining pharmacological treatment versus treatment withdrawal. The main outcome measures are institutionalization and/or functional disability. Table 1 summarizes the evaluations that will be used and Fig. 1 shows the schedule for enrollment, interventions, and assessments.

**Table 1:** Summary of visits and content

Instrument	Assessment	Timing of assessment
Inclusion form	Eligibility criteria	Previous to randomization
Sociodemographic form of patient and caregiver	Sociodemographic data	Basal
Baseline clinical data form	Treatment for dementia Diseases and concomitant treatments	Basal
Institucionalization form	Institutionalization date	1,3,6 & 12 months (study end)
SMMSE	Cognitive assessment	Basal, 1,3,6 & 12 months (study end)
BADLS	Functional assessment	Basal, 1,3,6 & 12 months (study end)
FAST	Functional assessment	Basal & 12 months (study end)
QUALID	Advanced dementia quality of life	Basal, 1,3,6 & 12 months (study end)
EQ-5D	Quality of life related to health	Basal & 12 months (study end)
NPI-Q	Psychological and behavioral symptoms associated to dementia	Basal, 1,3,6 & 12 months (study end)
Zarit Scale	Caregiver overload	Basal, 1,3,6 & 12 months (study end)
CGIC	Clinical improvement impression	12 months (study end)
RUD Lite	Use of health resources in dementia	12 months (study end)
Adverse effects forms	Safety, adverse effects and mortality	1,3,6 & 12 months (study end)

### Setting

We will perform this study in at least in 5 health centers and 1 hospital of the Balearic Islands (Spain), with the number determined by recruitment rate.

### Participants

#### Inclusion criteria

This study will include patients with advanced dementia who are living in the community and receiving treatment in a primary care setting or a hospital.

Participants must have the following criteria for enrollment:

1. Patient with dementia due to AD, according to National Institute on Aging and Alzheimer’s Association (NIA-AA) criteria [13], with or without small vessel subcortical vascular disease Fazekas 1 or 2, [14].
2. Advanced dementia (MMSE ≤10).
3. Use of drugs for dementia (a ChEI and/or memantine) in stable dose for 6 months or more.
4. Completion of informed consent agreement by the legal caregiver and the patient when appropriate.
5. Patients without clinical changes of dementia or acute decompensation of concomitant systemic diseases and stable in their pharmacological treatment for dementia or other diseases in the last 3 months

#### Exclusion criteria

Participants who exhibit any of the following will be excluded:

1. Patients with non-AD suspected pathology as the main cause of the dementia.
2. Life expectancy less than the follow-up duration of the study.
3. On a waiting list for interventions or treatments that require hospitalization.
4. Participating in another clinical trial.

### Randomization

A sequence of random numbers will be generated using the Epidat 3.1 program, and used for allocation to each of the two arms. After verification of patient eligibility, the recruiting researchers will make a telephone call to the research unit of the Primary Care Management to determine the group assignment. This allocation will occur after identification of the patient through an algorithm that assigns each patient to the control or intervention group using stratified block randomization. The two groups will be matched for mean durations of treatment with drugs for dementia prior to study onset (6 to 12 months vs. more than 12 months) and age (younger than 74 vs. 75 years or more). The randomization process will be recorded by collecting data from the code request date, patient identification code, and assigned treatment arm. The investigator who approves patient eligibility and requests the randomization will be different from the one who makes evaluations at the study visits. The main and secondary objectives of the study will be evaluated by health professionals who are not on the research team and who will remain blinded to group allocation. The person performing the statistical analysis will also be blinded to group allocation.

### Sample size

The total sample size for the primary objective, with a statistical power of 80%, an alpha error of 5%, and a 1:1 ratio of subjects in the two groups, is 251 patients. The assumptions for this calculation are: *(i)* the incidence of the main outcome measure (time to institutionalization and/or progression of the disability, defined as a loss of 2 of 4 basic functions, or 6 of 11 instrumental functions on the BADLS) is 25% at 12 months; *(ii)* the minimum HR for detection of a significant difference is 2.09; and *(iii)* the correlation between the studied variables is 0.002. Based on an assumed loss rate of 20% per year, at least 302 patients will be enrolled.

### Recruitment

Potential participants will be identified from the billing records of the Pharmacy of the Health Service of the Balearic Islands and the Neurology Service of the Hospital Son Espases from the previous year. After study onset, the list will be updated every 3 months to identify new candidates. Primary care health centers will be provided with a list of patients receiving treatment with drugs for dementia, and the recruitment will be based on review of their clinical histories. Billing information for 2015 in the Balearic Islands indicated there were 4169 patients over 75 years-old who received treatment with a drug for dementia for 3 months or more. We anticipate we may experience some difficulties in the recruitment due to the fact that the general practitioners participating in this study would be expected to discontinue a drug that was initially prescribed in the hospital setting, mostly by neurologists. Study visits will be performed whether at health care centers or at home to promote participant retention.

### Intervention

The intervention will be continuation or cessation of pharmacological treatment. This intervention will not require phased withdrawal, or any additional follow-up to assure patient safety. Adherence will be monitored through indirect adherence addressed by assessment of acquisition and possession of medication. This method is suitable to follow adherence in chronic treatment and when medication does not require frequent dosage changes [15].

### Primary outcomes

The main outcome measurement will be time to institutionalization and/or progression of disability (defined as a loss of 2 of 4 basic functions, or 6 of 11 instrumental functions using the BADLS). This scale was constructed as a functional assessment of patients with dementia who are living in the community. It is a simple scale that rates 20 basic and instrumental activities of daily life. Each item has 4 answers that evaluate the functional capacity. The total score varies from 0 (totally independent) to 60 (totally dependent) [16]. It is administered by health professional, has good inter- and intra-observer reproducibility, and good correlation with the Severe Mini-Mental State Examination (SMMSE) [17]. Data on the time to institutionalization will be collected by interview with the caregiver and/or review of the clinical history [18].

### Secondary outcomes

#### Functional assessment

Functional Assessment (FAST) scale will be used to assess daily life activities by examining functional loss in 16 categories that are related to self-care in patients with AD. The FAST scale is widely used in clinical practice as a tool to grade functional difficulties and monitor changes of patients over time [19].

#### Cognitive assessment

The SMMSE is a brief instrument used for rapid assessments, and does not require any special materials for administration. Thus, it can be used by different types of health care professionals to assess the cognitive status of patients This scale has 30 items that evaluate aspects of cognitive functions that remain somewhat preserved in patients with advanced AD: overlearned information, simple visual-spatial functions, executive functions, basic language functions, and simple semantic fluency [20].

#### Quality of life

The Quality of Life in Late-Stage Dementia (QUALID) scale collects information provided by the caregiver, based on repeated observations of the patient’s behavior during the previous week. It has 11 items that assess observable behaviors, which are indicative of the caregiver’s individual experience regarding the patient’s quality of life. These include subjective and affective states of the patient during daily life (smiling, crying, or seeming sad, annoying, irritable, or calm) and comfort or discomfort during basic activities of daily life from the social perspective (enjoyment of eating, touching, or interacting with others) [21].

The EuroQol five dimension (EQ-5D) scale measures self-perceived health, and incorporates individually perceived health status and is a recommended tool for use in cost-utility analyses. It consists of 2 parts: a descriptive section, determined in five dimensions, and an evaluation section, which uses a visual analog scale (VAS). The five dimensions of the descriptive section are: mobility, self-care, habitual activities, pain/discomfort, and anxiety/depression. In the evaluation section, the patient scores his/her health from 0 to 100 using a VAS [22].

#### Behavioral and psychological symptoms of dementia

The Neuropsychiatric Inventory-Questionnaire (NPI-Q) is administered to caregivers, who evaluate 12 aspects of the severity of symptoms in a patient. It is shorter than the NPI, and can be self-administered [23].

#### Clinical impression

The Clinical Global Impression of Change (CGIC) is a global classification of all domains of patient’s current disease, relative to the initial state. The score ranges from 1 (much better) to 7 (much worse), with 4 indicating no change. The doctor performs this evaluation at both time points, with contributions from family members and caregivers.

#### Cost-effectiveness

A cost-effectiveness analysis will be based on data collected from the *Resource Utilization in Dementia* (RUD) Lite scale, review of clinical histories, and costs of the drugs. Cost-effectiveness will be expressed in terms of quality-adjusted life years (QALY). The RUD Lite scale evaluates factors related to the direct and indirect costs of social and health care in patients with dementia. It determines the working status of the main caregiver, time spent helping the patient in the realization of basic and instrumental activities of daily life, lost work hours, consumption of drugs, and number of visits with the patient to social and health care services [24].

#### Caregiver overload

The Zarit scale is an instrument that quantifies the extent of “overload” experienced by caregivers of dependent people. It consists of a list of 22 items that describe how caregivers feel. For each item, the caregiver indicates the frequency of an event, using a scale from 0 (never) to 4 (almost always) [25].

#### Safety

Mortality, AEs, and complications associated with dementia (emergency room visits and/or *hospitalization*) will be collected throughout the study.

### Independent variables

The main independent variable is membership in the intervention group or control group.

#### Independent variables

The additional independent variables are:

a) Socio-demographic characteristics (age, sex, marital status, having a formal or informal caregiver).b) Type of previous pharmacological treatment for dementia.c) Duration of previous treatment (3 to 6 months vs. more than 6 months).

### Statistical analysis

#### Descriptive analysis, labeling, and filtering of data

Assessment of atypical values and outliers, detection and labeling of lost and/or non-applicable values, and the distribution of variables will be assessed using normality tests and scatter plots.

#### Baseline comparative analysis

Comparison of the socio-demographic characteristics of the two groups will use Student’s *t*-test or the Chi-square test (where appropriate). Non-parametric tests will be used when the data have non-normal distributions.

#### Final comparative analysis

All analyses will be performed using the intention-to-treat principle, with a two-sided level of statistical significance of 5% and adjustment for the stratification criteria of the randomization. The main outcome variable — time to institutionalization and/or progression of disability — will be assessed using Cox regression. A sensitivity analysis will be used to analyze raw efficacy data (log-rank test), Cox regression data adjusted for non-homogeneous variables, and stratification variables. The nonparametric Mann-Whitney U test and the Chi-square test will be used for analysis of the scales used for secondary outcome measures and for analysis of safety data.

### Cost-effectiveness analysis

The incremental cost-effectiveness ratio (ICER) will be calculated by dividing the difference in costs by the difference in effectiveness of the two groups. To estimate the level of uncertainty in the estimates of cost differences and the ICER, bootstrapping with 500 replications of each data set will be performed. Due to the skewed distributions of costs, a correction will be performed using the bias-corrected and accelerated method. The estimated costs (determined by bootstrapping) and efficiency will be expressed in graphs and acceptability curves. The cost-effectiveness, net benefit, and incremental net benefit will also be calculated.

### Validation of the BADLS and QUALID scales

A psychometric approach will be used to determine the extent to which the instruments measure what they intend to measure in the selected sample, to assure they reach minimum conditions of validity and reliability. The properties to be examined are: *(i)* viability/acceptability of the data (percentage of lost data, range of observed and possible scores, measures of central tendency and dispersion, ground and ceiling effects, asymmetry); *(ii)* reliability (internal consistency using Cronbach’s alpha coefficient, coefficient of homogeneity of the items and correlations between items, test-retest reliability using the correlation coefficient); *(iii)* validity (discriminative validity using Student’s *t*-test, ANOVA, Mann-Whitney U test, or Kruskal-Wallis test; convergent validity using the correlation coefficient; divergent validity using the correlation coefficient; internal validity using factor analysis and inter-domain correlations; and criterion validity using correlation coefficients); and *(iv)* precision of measurements using standard error of mean (standard deviation/√n).

### Data collection, management, and analysis

Each participant will be identified by a study number only, and the master code sheet linking names with numbers will be held securely and separately from the study data. To ensure that all information is secure, data records will only be accessible to research staff and authorized personnel. After completion of all follow-ups, the data records will be de-identified and the de-identified data will be used for statistical analysis. All resulting publications will only include aggregate data.

Data entry will be done with Opentext TeleForm, a software that automatically capture, extract data and validate from paper reported form. It enables a double data entry and range checks for data values.

### Monitoring

The trial will be audited by monitor at the beginning and at the end of the study. The process will be independent from the investigators and the sponsor.

Adverse events will be reported during this trial will be collected, assessed and reported as indicated in EU directive 2001/20/EC.

## Discussion

Previous studies on the efficacy of pharmacological interventions for dementia have been limited by numerous factors, such as short study duration, failure to include patients with severe AD, inadequate reporting of adverse events, failure to use clear definitions in reporting clinical significance, inadequate evaluation of behavior and quality-of-life outcomes, and inadequate direct comparisons of different treatments. Thus, there is no consensus regarding which outcomes are most clinically relevant during different stages of dementia. Furthermore, the magnitude of a clinically relevant change may depend on whether the change is reported by the patient, the caregiver, or the clinician [26].

Previous clinical trials that evaluated the clinical efficacy of anti-dementia drugs have mainly examined patients with AD, and excluded patients with other types of dementia. In addition, despite the high prevalence of dementia, most clinical trials that examined patients with severe dementia only enrolled small numbers of patients, with the exception of the DOMINO-AD which examined 154 patients. Most previous assessments of efficacy have focused on cognitive performance, but this may be the incorrect focus for patients with advanced dementia because it does not consider the needs of the patients and their families. For patients with moderate-to-severe dementia, there is an increasing emphasis on preserving function (i.e., activities of daily life), delaying institutionalization, and management of disruptive behaviors, all of which are burdens for the caregiver [27].

To overcome the limitations of previous studies, the main outcome measure of the present study is time to institutionalization and/or progression of disability evaluated using the BADLS [6]. Although some previous studies of patients with dementia have examined time to institutionalization, patients must often wait for admission to public centers, and its assessment is non-viable, so it has been chosen to add the assessment of the progression of the disability. Moreover, the advantages of the BADLS are that it evaluates basic and instrumental functions, it has good sensitivity for patients with advanced dementia.

We used an open clinical trial design, in which only the evaluators will be blinded. Analysis of the use of anti-dementia drugs from our region indicates that even though 45% of the drugs given to those who are more than 75 years-old are in tablet form, up to 44% of patients used other formulations to improve dosing (transdermal patches, orodispersible tablets, or oral solutions). Maintaining masking by use of placebo tablets could compromise recruitment. For this reason we used a robust main outcome measure — institutionalization and/or functional impairment using the BADLS — with blinded evaluators. Despite the pragmatic approach of our study, many patients who have dementia due to Parkinson’s disease, Lewy bodies, or multiple infarctions receive treatment with the same drugs. Thus, we excluded these individuals because their quality of life, health status, and survival does not depend only on their cognitive and functional status. Moreover, experts agree that treatment of dementia is complex, and the use of different pharmacological agents requires detailed observations and assessments to assess potential adverse effects [28].

The present pragmatic clinical trial, which will only assess patients with severe dementia due to AD, with or without small vessel subcortical vascular disease and will compare the efficacy of maintaining pharmacological treatment vs. treatment cessation on the time to institutionalization and/or functional disability. Assuming that a patient’s quality of life is better at home than at a nursing home, we consider nursing home admission as an outcome measure itself, because nursing home admission is considered an event to be delayed for as long as possible, from perspectives of patients and their families [29, 30].

We hope that our trial will contribute to provide more evidence on whether or not deprescribing and the feasibility of the withdrawal of these drugs in patients with advanced stage of dementia.

## Abbreviations

AD: Alzheimer’s disease
AE: Adverse event
BADLS: Bristol Activities of daily living scale
CGIC: Clinical Global Impression of Change
ChEI: Cholinesterase inhibitor
EQ-5D: EuroQol five dimension
FAST scale: Functional Assessment s*cale*
HR: Hazard Ratio
ICER: Incremental cost-effectiveness ratio
MMSE: Mini-Mental State Examination
NIA-AA: National Institute on Aging and Alzheimer’s Association
NMDA: N-methyl D-aspartate
NPI-Q: Neuropsychiatric Inventory-Questionnaire
QALY: Quality-adjusted life years
QUALID: Quality of Life in Late-Stage Dementia
RR: Relative risk
RUD: *Resource Utilization in Dementia*
SMMSE: Severe Mini-Mental State Examination
SPBD: Symptoms of Psychological and Behavioral Dementia
VAS: Visual analog scale
VD: Vascular dementia
